# Epidemiological alteration in pathogens found in ground meat in Iran: unexpected predominance of vancomycin-resistant Enterococcus faecalis

**DOI:** 10.3205/dgkh000255

**Published:** 2015-07-14

**Authors:** Nourkhoda Sadeghifard, Hossein Kazemian, Reza Mohebi, Zamberi Sekawi, Afra Khosravi, Nasrin Valizadeh, Sobhan Ghafourian

**Affiliations:** 1 Clinical Microbiology Research Center, Ilam University of Medical Sciences, Ilam, Iran; 2 Department of Medical Microbiology and Parasitology, Faculty of Medicine and Health Sciences, Universiti Putra Malaysia, Serdang, Malaysia

**Keywords:** Enterococcus faecalis, VRE, Escherichia coli O157H7, ground meat, food, Iran

## Abstract

Colonization of the human and animal intestinal tract with potential pathogenic bacteria is correlated with the risk of contamination of food products. The current study analyzed the prevalence of *Enterococcus faecalis* and *Escherichia coli* O157H7 in ground meat in Ilam, Iran.

Both index organisms were identified following standard food microbiological methods. For *E. faecalis*, the susceptibility to vancomycin was tested, and PCR was used to check for the *vanA* gene.

*E. faecalis* was present in all 24 ground meat samples, with no *E. coli* O157H7 detected in samples. The analysis showed the presence of the *vanA* gene in 5/24 vancomycin resistant enterococci.

In conclusion, this study for the first time demonstrates the presence of vancomycin-resistant enterococci in ground meat in Iran. This observation warrants further epidemiologic investigation and should be followed up in the future.

## Zusammenfassung

Das Vorkommen potentiell pathogener Bakterien im menschlichen und tierischen Darmtrakt ist mit einem Kontaminationsrisiko für Lebensmittel verbunden. Daher wurde die Prävalenz von *E. faecalis* und *Escherichia coli* O157H7 in Hackfleisch in Ilam, Iran, untersucht.

Nach der Probennahme wurden die Bakterien gemäß lebensmittelhygienischer Methode untersucht. Für *E. faecalis* wurde ihre Sensitivität gegenüber Vancomycin geprüft und das Vorkommen des *vanA*-Gens mittels PCR untersucht.

In allen 24 Hackfleischproben war *E. faecalis*, jedoch in keinem Fall *E. coli* O157H7 nachweisbar. In 5 der 24 untersuchten Lebensmittelproben wurde VRE-Stämme mit Nachweis des *vanA*-Gens nachgewiesen. Diese Studie zeigt erstmals das Vorkommen von VRE in Hackfleisch im Iran. In Anbetracht des Vorkommens von VRE sollten weitere epidemiologische Untersuchungen durchgeführt und die Entwicklung genau beobachtet werden.

## Introduction

Negligence of safe food handling, particularly beef and lamb, among the majority of the Iranian population causes high frequencies of chronic disease, including food-related diarrhea [1]. Meat contaminated with *Escherichia coli* O157H7 ranks among the most severe food-related diarrheal diseases [2]. However, a less pathogenic, but epidemiologically relevant intestinal pathogen is *Enterococcus faecalis*, which lives commensally in both human and animal intestinal tracts. If food is handled inappropriately, contamination of food products may be the consequence [3]. During the past few years, food products contaminated with vancomycin-resistant enterococci (VRE) have been reported worldwide, and are responsible for morbidity and mortality among hospitalized patients or those receiving systemic antibiotic treatment [3]. To date, no study exists on the prevalence of *E.coli* O157H7 and *E. faecalis* in meat in Iran. Therefore, a pilot study was conducted to analyze the prevalence of both index organisms in ground meat.

## Methods

To investigate the presence or absence of *E. coli* O157H7 and/or *E. faecalis* in meat, 24 samples of ground, not cooked meat were collected in 14 “kebab” restaurants in Ilam, Iran. Additionally, 10 samples of raw meat were obtained and screened. Index organisms were identified by conventional microbiological methods and biochemical tests. If *E. faecalis* was found, minimal inhibitory concentration to vancomycin was determined following CLSI recommendations. Additionally, a *vanA*-specific PCR was performed to verify vancomycin-resistant enterococci (VRE) strains.

## Results

**Figure 1:**
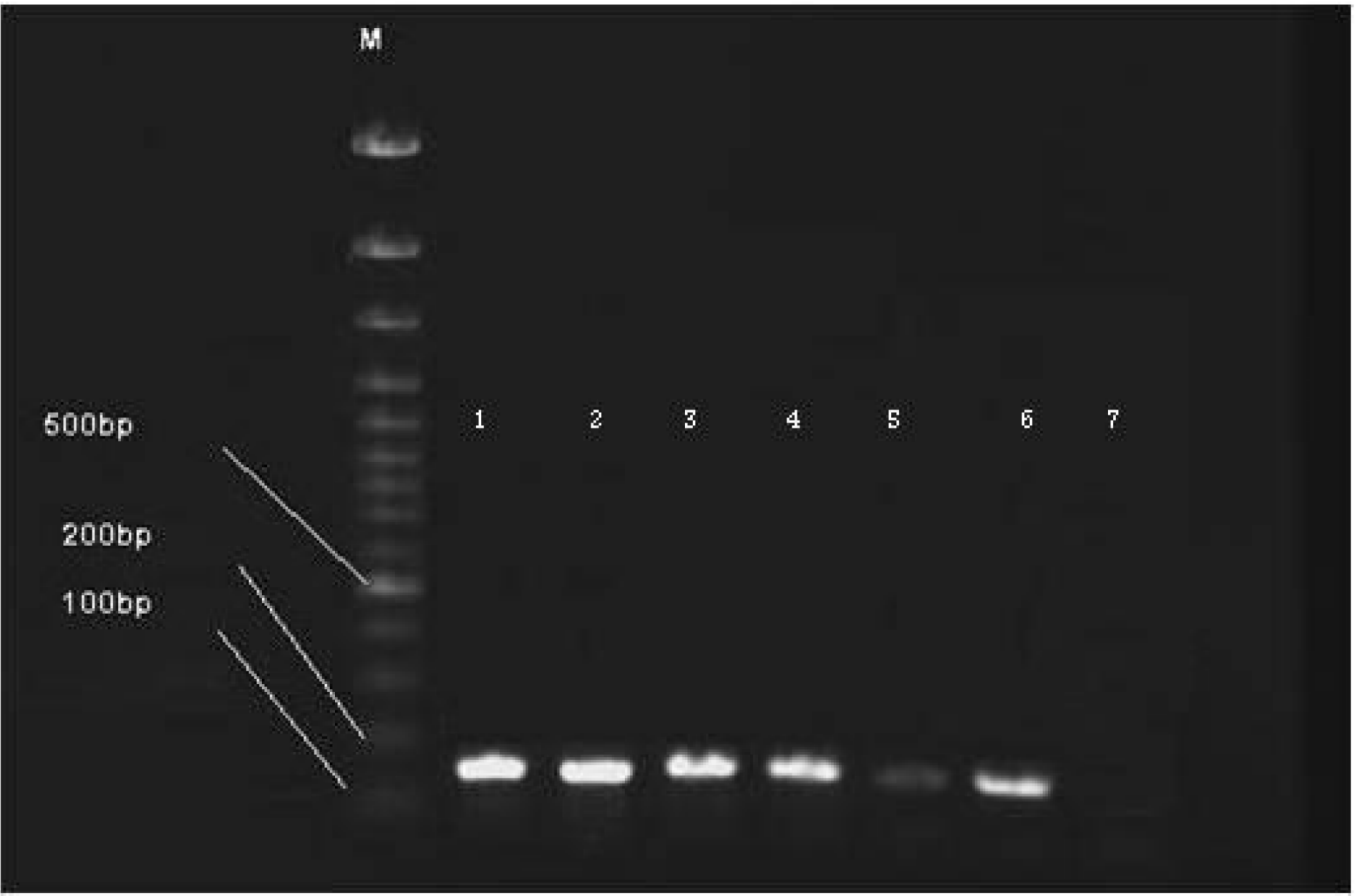
*vanA E. faecalis*; M = Marker (100bp); 1 = positive control; 2–6 = *vanA* positive strains; 7 = negative control

While a few previously conducted studies [4], [5] reported on the presence of *E. coli* O157H7 without detecting VRE, the present investigation found the opposite result. Although we observed no *E. coli* O157H7 strains among 24 ground meat samples, *E. faecalis* was obtained from all 24 samples of ground, cooked kebab meat and 10 raw meat samples. Furthermore, all *E. faecalis* isolates showed resistance to vancomycin, with 5 strains also showing the presence of the *vanA* gene in the plasmid [6] of *E. faecalis* isolates (Figure 1 (Fig. 1)). This is the first report of VRE presence in ground meat in Iran.

## Discussion

To date, most studies have considered *E. coli* O157H7 as the most important pathogenic microorganism in ground meat in Iran [1], [2]. Surprisingly, none of the ground meat samples investigated in this study was contaminated by *E. coli* O157H7, whereas *E. faecalis*, including VRE, was found in ground meat in Iran. This study is the first study reporting on *E. faecalis*, and particularly VRE, in ground meat in Iran.

Because *E. faecalis* is a constituent of the gut microflora in animals and humans [3] with a close association between humans and farm animals, enterococci were identified as contaminants in meat [3], [7]. The occurrence of 5 positive *vanA* genes in *E. faecalis* isolates are of particular interest, since VRE is associated with nosocomial infections. Although kebab in Iran is well cooked, the presence of *E. faecalis* and VRE in our tested ground meat is of epidemiological interest.

## Conclusion

Our observation warrants further epidemiologic investigation and should be studied in greater depth in the future. Furthermore, restaurants should be monitored more closely to control critical bacteria in animal products, especially in ground meat.

## Notes

### Competing interests

The authors declare that they have no competing interests.

## References

[1] Azadbakht L, Rouhani MH (2012). Red meat consumption: Emphasis on chronic diseases or sticking to nutrient deficiency?. J Res Med Sci.

[2] Zhao C, Ge B, De Villena J, Sudler R, Yeh E, Zhao S, White DG, Wagner D, Meng J (2001). Prevalence of Campylobacter spp., Escherichia coli, and Salmonella serovars in retail chicken, turkey, pork, and beef from the Greater Washington, D.C., area. Appl Environ Microbiol.

[3] Animal and Plant Health Inspection Service, Veterinary Services, Centers for Epidemiology and Animal Health (2014). Enterococcus on U.S. Sheep and Lamb Operations.

[4] Centers for Disease Control and Prevention (2014). Multistate Outbreak of Shiga toxin-producing Escherichia coli O157:H7 Infections Linked to Ground Beef (Final Update).

[5] Doyle MP, Schoeni JL (1987). Isolation of Escherichia coli O157:H7 from retail fresh meats and poultry. Appl Environ Microbiol.

[6] Sadeghifard N, Soheili S, Sekawi Z, Ghafourian S (2014). Is the mazEF toxin-antitoxin system responsible for vancomycin resistance in clinical isolates of Enterococcus faecalis? GMS Hyg Infect Control. http://dx.doi.org/10.3205/dgkh000225.

[7] Sparo MD, Confalonieri A, Urbizu L, Ceci M, Bruni SF (2013). Bio-preservation of ground beef meat by Enterococcus faecalis CECT7121. Braz J Microbiol.

